# The Role of Xenobiotic Caffeine on Cardiovascular Health: Promises and Challenges

**DOI:** 10.3390/jox15020051

**Published:** 2025-03-31

**Authors:** Roberto Campagna, Arianna Vignini

**Affiliations:** 1 Department of Clinical Sciences, Polytechnic University of Marche, 60100 Ancona, Italy; 2 Research Center of Health Education and Health Promotion, Polytechnic University of Marche, 60100 Ancona, Italy

**Keywords:** caffeine, 3,7-dihydro-1,3,7-trimethyl-1H-purine-2,6-dione, cardiovascular diseases

## Abstract

Cardiovascular diseases (CVDs) represent a leading cause of premature mortality and disability worldwide, with their incidence expected to rise, potentially reaching 24 million deaths per year by 2030. These multifactorial diseases, including hypertension, coronary artery disease, arrhythmia, and heart failure, are often linked to metabolic disturbances such as diabetes, oxidative stress, endothelial dysfunction, and inflammation. Natural compounds, such as caffeine, have been explored for their potential therapeutic effects on CVDs. Caffeine, found in coffee, tea, cocoa, and various energy drinks, is a widely consumed psychoactive compound with noted analgesic and anti-inflammatory properties. Despite its long history of use, caffeine’s impact on cardiovascular health remains controversial, with both beneficial and harmful effects reported. This review examines the current literature on the effects of caffeine on cardiovascular diseases (CVDs), with an emphasis on preclinical and clinical studies, its pharmacokinetic properties, and the molecular mechanisms it modulates. There is evidence that moderate caffeine intake can be beneficial for some CVDs, such as hypertension, while for other CVDs, such as dyslipidemia, the evidence collected so far suggests that caffeine intake could be detrimental since it increases total cholesterol levels. But variability in dosage, intake patterns, and individual factors (such as genetics and diet) complicates the reliability of results. Additionally, challenges related to dose standardization and the absence of consistent clinical trial designs hinder the full utilization of caffeine in CVD treatment. Nonetheless, caffeine appears to be safe for individuals without significant cardiovascular conditions. Future research should aim for well-designed studies with precise patient cohorts and standardized methodologies to better assess caffeine’s role in CVD management.

## 1. Introduction

Cardiovascular diseases (CVDs) are a variegated group of disorders involving the heart and vasculature that collectively represent the primary cause of premature mortality and disability in humans globally [1]. Indeed, it is estimated that the incidence of CVDs will constantly increase, reaching a value of 24 million deaths per year by 2030 [2]. CVDs are complex and multifactorial diseases and include hypertension, coronary artery disease, arrhythmia, cardiomyopathy, peripheral arterial disease, and heart failure [3]. CVDs physiopathology is particularly complex and often involves metabolic disorders, such as diabetes, oxidative stress, endothelial dysfunction, and inflammation [4,5,6,7,8,9,10,11]. Despite ongoing advancements in the medical field, including the discovery of novel bioactive compounds and improvements in diagnostic and therapeutic strategies, CVDs remain the leading cause of mortality in developed countries, thereby posing a substantial economic burden on healthcare systems [12].

Natural compounds play a key role in counteracting the onset and occurrence of several cancerous and non-cancerous diseases [13,14,15,16,17,18,19,20,21]. Caffeine (also known as 3,7-dihydro-1,3,7-trimethyl-1H-purine-2,6-dione) (CAF) is a natural compound that can be found in coffee, tea, guarana, and cocoa and has been largely studied for its potential beneficial effects in CVD management. Indeed, beyond being a very common psychoactive compound present in several food and beverages, such as cocoa, chocolate, coffee, tea, sodas, and energy drinks, it is also a component of several drugs due to its analgesic and anti-inflammatory properties [22]. Due to the large consumption of caffeinated food and beverages worldwide, CAF is considered the most representative pharmaceutically active xenobiotic in the environment [23,24,25,26]. Though CAF has been commonly used since ancient times, it is still a controversial molecule and the object of many studies. The studies published in the literature regarding this bioactive molecule convey both the beneficial effects and toxic effects on human health. This review offers a comprehensive overview of the existing literature on studies investigating the effects of CAF on CVDs. It also discusses the pharmacokinetics of caffeine, the molecular mechanisms it modulates, and the challenges and limitations that have hindered its application as a therapeutic agent for CVDs so far. Finally, we provide a suggestion about how further studies should be designed in order to definitely elucidate the role of caffeine in CVDs.

## 2. Metabolism of Caffeine

### 2.1. Sources, Metabolism, and Pharmacokinetics of Caffeine

**Figure 1:**
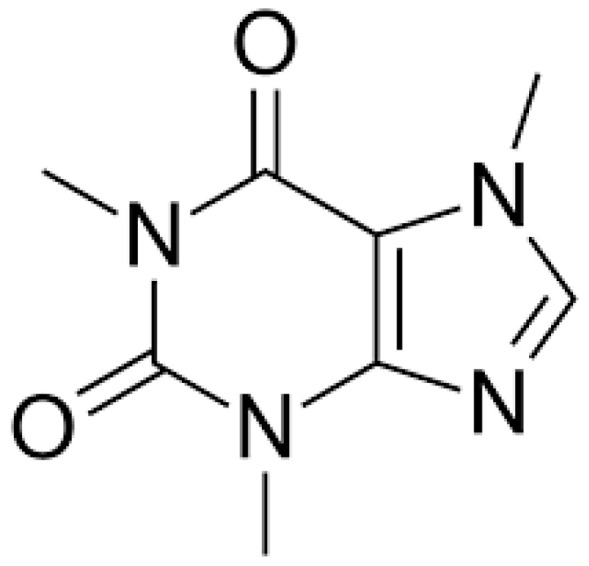
Caffeine (1,3,7-trimethylxanthine) chemical structure.

CAF (1,3,7-trimethylxanthine), the chemical structure of which is reported in Figure 1, is a xenobiotic alkaloid for humans, which can be synthetically produced but is also naturally found in many vegetables, especially in beans, leaves, and fruits. Although roasted coffee beans (Coffea Arabica and Coffea robusta) and tea leaves (Camelia siniensis) are the primary sources of dietary CAF worldwide, there are also many other common sources, such as the kola nut (Cola acuminate), cacao bean (Theobroma cacao), yerba mate (Ilex paraguariensis), and guarana berries (Paullinia cupana) [27]. The bulk of CAF consumption is represented by beverages such as coffee, soft drinks, and tea, as reported by the European Food Safety Authority (EFSA), and the average daily consumption of CAF in young adults (18–65 years old) is estimated to be 37–319 mg [28]. However, the consumption of CAF is progressively increasing due to the constant release in the market of novel functional beverages, including energy drinks and other caffeinated beverages, such as sports drinks, juices, and flavored waters [24,29,30].

**Figure 2:**
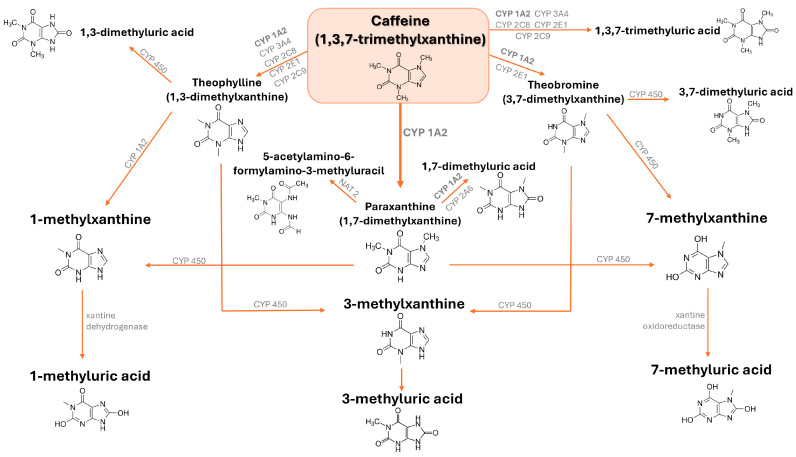
Caffeine (1,3,7-trimethylxanthine) metabolic pathways.

Upon ingestion, CAF is promptly absorbed by the gastrointestinal tract and, through the bloodstream, it arrives in the liver, where it is metabolized into three main intermediate metabolites named 1,7-dimethylxanthine (paraxanthine) for 80%, 3,7-dimethylxanthine (theobromine), and 1,3-dimethylxanthine (theophylline) to a smaller extent [31,32]. Up to 100 mg of CAF exhibits a linear pharmacokinetics, while for doses of 250 to 500 mg, the CAF clearance is significantly diminished and its elimination half-life is prolonged, implying nonlinearity [33,34]. Theophylline is further processed into 1-methylxanthine or 3-methylxanthine, which can be finally demethylated and oxidized into 1-methyluric acid or 3-methyluric acid, respectively. Paraxanthine is processed into 1-methylxanthine or 7-methylxanthine, which are finally demethylated and oxidized into 1-methyluric acid or 7-methyluric acid, respectively, while theobromine is first converted to 3-methylxanthine or 7-methylxanthine before being demethylated and oxidized to 3-methyluric acid or 7-methyluric acid, respectively (Figure 2) [35]. Around 90% of the CAF of a single cup of coffee is eliminated from the stomach within 20 min, with peak plasma concentration occurring roughly 1 to 1.5 h later [31]. Beyond the oral administration, CAF can be administered intravenously or through the rectal route, as citrate, sodium benzoate, or ergotamine tartrate pharmaceutical salts. Since CAF is a water-soluble compound with hydrophobic properties and low protein binding (10–35%), it easily enters and distributes within cells and tissues, being able to also penetrate the blood-brain barrier effectively [36].

Importantly, CAF metabolism varies among individuals. A recent study involving 2278 participants found that terminal caffeine metabolites, including urinary methyl uric acids and methylxanthines, significantly lower the risk of hypertension [37]. After absorption, CAF produces various physiological effects on different organs of the body. At the typical doses found in coffee, tea, and soft drinks, its primary mechanism of action is acting as an adenosine receptor antagonist in the brain, leading to inhibitory effects on the central nervous system [38,39]. Indeed, due to its high structural similarity to adenosine, CAF is able to localize into adenosine receptor sites, primarily A_1_ and A_2a_ [40,41,42,43]. Upon binding to adenosine receptors, caffeine blocks the ability of adenosine to slow nerve activity, causing the neurons to become more active rather than slowing down [43,44].

### 2.2. Genetic Modifiers of Caffeine Metabolism

Several studies have demonstrated that CYP1A2 polymorphisms influence caffeine metabolism and clinical outcomes from caffeine intake [45,46]. Womack et al. reported that the A (C/A) single nucleotide polymorphism at intron 1 of the cytochrome P450 (CYP1A2) gene influences caffeine metabolism, as subjects homozygous for the A allele of this polymorphism exhibit a boosted ergogenic effect following caffeine intake [47]. Thomas et al. investigated how a CYP1A2*1F polymorphism affects post-exercise heart rate variability (HRV) in response to caffeine consumption in a cohort composed of A/A homozygotes or C allele carriers. Although no significant differences emerged in HRV indices, the square root of the mean of squared differences between successive RR intervals was the sole variable affected by the CYP1A2*1F polymorphism after exercise following caffeine consumption [48]. Cornelis et al. investigated whether the CYP1A2 genotype influences the association between coffee intake and risk of acute nonfatal myocardial infarction [49]. Indeed, subjects who are homozygous for the CYP1A21A allele are known to quickly metabolize caffeine, while individuals with the CYP1A21F variant are known to be slow caffeine metabolizers [50,51,52]. Coffee consumption was linked to a higher risk of nonfatal myocardial infarction in subjects with slow caffeine metabolism, indicating that caffeine may contribute to this relationship [49]. Minaei et al. examined the impact of the CYP1A2 -163C > A polymorphism on anaerobic power in trained males upon CAF administration. Consuming 6 mg/kg of caffeine enhanced peak power output exclusively in subjects carrying the AA genotype compared to the placebo [53]. Guest et al. reported that caffeine boosts endurance performance at doses of 2–4 mg/kg in fast caffeine metabolizers characterized by the CYP1A2 AA genotype [54].

Recent research examining genetic variations in both animal models and humans has identified polymorphisms in the adenosine A_1_ and A_2A_ receptors as key factors influencing the physiological response to caffeine. Experimental animal studies have demonstrated that A_2A_ receptors play a critical role in modulating the reinforcing behavioral effects of caffeine, as well as its impact on the regulation of the sleep cycle, which is well known to be correlated with CVD risk [55]. Furthermore, emerging evidence from human studies suggests that distinct polymorphisms in the A_2A_ receptor gene are linked to alterations in caffeine-induced anxiety and sleep disturbances, particularly in individuals with heightened sensitivity to caffeine. Notably, anxiety is considered an important risk factor for CVDs [56,57,58]. The adenosine A_2A_ receptor is implicated in mediating the arousal effects induced by caffeine. In experiments with mice lacking functional A_2A_ receptors, there is an absence of the typical increase in wakefulness following caffeine administration, suggesting that the A_2A_ receptor is essential for the caffeine-induced arousal response [59]. Interestingly, in humans, the rs5751876 polymorphism in the A_2A_ receptor has been linked to disruptions in sleep and heightened beta band activity in the electroencephalogram (EEG) following caffeine consumption [60]. The ADORA2A rs5751876 C/C genotype (1976 C→T, formerly referred to as 1083 C→T) was more commonly detected in individuals who identified themselves as caffeine sensitive, while the T/T genotype was more prevalent in those who reported being caffeine insensitive. Additionally, individuals who considered themselves caffeine sensitive also experienced a higher incidence of caffeine-induced sleep disturbances, and this association between caffeine sensitivity and sleep disruption was further supported by EEG findings, which revealed increased beta activity during non-REM sleep in C/C genotype carriers, a pattern commonly observed in patients with insomnia [61,62]. On the contrary, individuals with the C/T genotype exhibited only half the increase in beta activity compared to those with the C/C genotype, while no change was observed in the T/T genotype, suggesting that the rs5751876 genotype influences the likelihood of developing caffeine-induced insomnia. Importantly, this correlation was independent of anxiety despite caffeine-sensitive individuals who reported higher anxiety levels. Although anxiety can contribute to insomnia, it was not found to correlate with the ADORA2A genotype in this investigation. Studies involving humans have indicated that polymorphisms in the A_2A_ receptor may contribute to adverse responses to caffeine in certain subjects. Specifically, the ADORA2A SNPs rs5751876 and rs35320474 (2592 T/−) have been linked to increased anxiety in low-caffeine consumers. Individuals carrying the rs5751876 T/T genotype, as well as those with the rs35320474 T/T allele, reported higher levels of anxiety following acute caffeine consumption compared to other groups [63]. A follow-up study involving low-caffeine users confirmed this positive association, although this correlation was no longer statistically significant when the analysis was limited to Caucasian subjects [64]. The study also identified two additional SNPs in ADORA2A, rs2298383 and rs4822492, as being associated with caffeine-induced anxiety. Notably, two distinct alleles at the rs5751876 locus were linked to different caffeine-related effects in Caucasian subjects: the C allele was associated with sleep disturbances induced by caffeine, while the T allele was linked to increased anxiety [60,63]. The associations between caffeine-induced anxiety and ADORA2A polymorphisms are particularly noteworthy when considered in the broader context of studies linking ADORA2A to drug-induced anxiety and anxiety disorders. Specifically, both the rs5751876 C/T and rs35320474 T/− polymorphisms have been associated with heightened anxiety following the acute administration of amphetamine in healthy individuals [65]. The rs5751876 T/T allele has been linked to panic disorder in Caucasian populations, although this association was not replicated in studies involving Japanese and Chinese populations, and this suggests that these genotypes may influence not only caffeine-induced anxiety but also general anxiety and anxiety disorders in specific populations [66,67,68,69]. The fact that the same SNP is associated with both caffeine-induced anxiety and panic disorder reinforces the observation that individuals with panic disorder are particularly vulnerable to caffeine-induced anxiety, indicating that polymorphisms in the A_2A_ receptor may play a role in both conditions [70]. It has been reported that A2A receptors are also implicated in the reward caffeine-induced effects. Studies with A2A knockout mice showed reduced caffeine self-administration compared to wild-type mice, indicating an important role for A2A receptors in the reinforcing properties of caffeine [71]. A cross-sectional study examining the relationship between ADORA2A polymorphisms and caffeine consumption provides further support for the involvement of A2A receptors in the negative reinforcement properties of caffeine in humans. In a study of Costa Rican individuals without a history of hypertension, those with the rs5751876 T/T genotype were found to consume less caffeine than individuals with the C/C genotype [49]. Nonetheless, the study did not assess anxiety, which could influence caffeine consumption and has been linked to the rs5751876 T/T genotype, as mentioned earlier. Epidemiological studies further suggest that anxiety may play a role in caffeine consumption, as individuals with panic disorder tend to consume less caffeine than those without a history of the disorder [72]. Taken together, this evidence suggests that genetic polymorphisms may greatly influence caffeine responses, which can impact risk factors for CDVs. Additional studies are required to better clarify the impact of these polymorphisms in the context of specific CVD.

## 3. Cardiovascular Benefits of Caffeine

### 3.1. Hypertension

Hypertension is a chronic medical condition characterized by persistent high blood pressure in the arteries [73]. Although high blood pressure does not often produce noticeable symptoms, it significantly increases the risk of stroke, coronary artery disease, heart failure, atrial fibrillation, peripheral artery disease, vision loss, chronic kidney disease, and dementia, thus being a leading cause of premature death globally [74]. A number of studies have been performed in order to investigate the effects of CAF on blood pressure (Table 1). A study performed by Oboh et al. investigated the effect of single or combined administration of CAF and caffeic acid on the activities of arginase, angiotensin-1-converting enzyme (ACE), nitric oxide (NO), and malondialdehyde (MDA) levels in L-NAME-induced hypertensive rats. CAF and caffeic acid combinations exerted antihypertensive effects by reducing systolic blood pressure, associated with decreased ACE and arginase activity and high NO and low MDA levels, suggesting that the combination of these molecules displays the potential for possible use in the management of hypertension [75]. Although these findings were interesting, their valuation should take into account the small size group of rats involved in this study. Noordzij et al. performed a meta-analysis of 16 studies with a randomized controlled design, which involved a total of 1010 subjects. The study concluded that drinking coffee (725 mL/day) or administering CAF (410 mg/day) leads to an increase in systolic pressure of 2.04 mmHg (95% confidence interval (CI) 1.10–2.99), while diastolic blood pressure was increased by 0.73 mmHg (95% CI 0.14–1.31) [76]. The Singapore Chinese Health Study attempted to deepen the relationship between coffee and tea and the risk of hypertension. In this population-based prospective cohort study, 63,257 Chinese aged 45–74 years stably living in Singapore were recruited. The researchers collected information on coffee and tea consumption at baseline, together with other lifestyle factors, and hypertension diagnosis was assessed during two follow-up timelines. In this study, an inverse U-shaped correlation emerged between the quantity of coffee intake and blood pressure alterations. The consumption of 1 cup of coffee/week (hazard ratio (HR) 0.87, 95% CI 0.83–0.91) or more than 2 cups/day (HR 0.93, 95% CI 0.86–1.00) triggered a decrease in hypertension risk compared with consuming 1 cup of coffee/day [77]. D’Elia et al. performed a random-effects dose-response meta-analysis of four studies, which included a total of 196,256 participants, of whom 41,184 were already diagnosed with hypertension. The results of this study showed a non-linear relationship between coffee intake and the risk of hypertension. Although 1–2 coffee cups intake per day was not associated with a risk of hypertension compared to non-drinking, the consumption of 3 cups/day showed a significant protective effect (relative risk 0.97, 95% confidence interval 0.94–0.99), and this effect was also confirmed in cases of greater consumption, thus suggesting that moderate coffee intake is not associated with a higher risk of hypertension and that a non-linear inverse dose-response relationship occurs between coffee intake and the risk of hypertension [78]. A clinical trial involving 48 men performed by Pincomb et al. was conducted to explore whether vasoconstrictive actions of CAF are enhanced in hypertensive patients. The authors found that an intake of 3.3 mg/kg of CAF is associated with a significant increase in diastolic blood pressure in patients with borderline hypertension compared with the controls (+8.4 vs. +3.8 mmHg, *p* = 0.0001) [79]. Hartley et al. investigated the acute effects of caffeine on arterial blood pressure in 5 hypertension risk groups for a total of 182 recruited men in order to verify whether patients with hypertension could be more susceptible to CAF. Subjects were classified into five hypertension risk groups according to the JNC VI16 criteria: optimal systolic blood pressure (SBP) 120 mmHg and diastolic blood pressure (DBP) 80 mmHg; normal SBP 120 to 129 mmHg or DBP 80 to 84 mmHg; high-normal SBP 130 to 139 mmHg or DBP 85 to 89 mmHg; stage 1, SBP 140 to 159 mmHg or DBP 90 to 99 mmHg; and diagnosed hypertension. Blood pressure was measured during CAF testing after 20 min of rest and once more at 45 to 60 min after the oral administration of CAF 3.3 mg/kg or a fixed dose of 250 mg. In this study, non-hypertensive subjects did not reveal any impact on blood pressure, whereas significant increased blood pressure was detected in patients at stage 1 hypertension [80]. Teramoto et al. conducted a study to examine the impact of coffee consumption on CVD mortality among patients affected by severe hypertension involving 6574 men and 12,035 women. Subjects were divided into the following groups: diagnosed hypertension, stage 1 (systolic blood pressure [SBP] 140–159 mmHg or diastolic blood pressure [DBP] 90–99 mmHg), high-normal (SBP 130–139 mmHg or DBP 85–89 mmHg), normal (SBP 120–129 mmHg or DBP 80–85 mmHg), and optimal (SBP < 120 mmHg and DBP < 80 mmHg).

**Figure 3:**
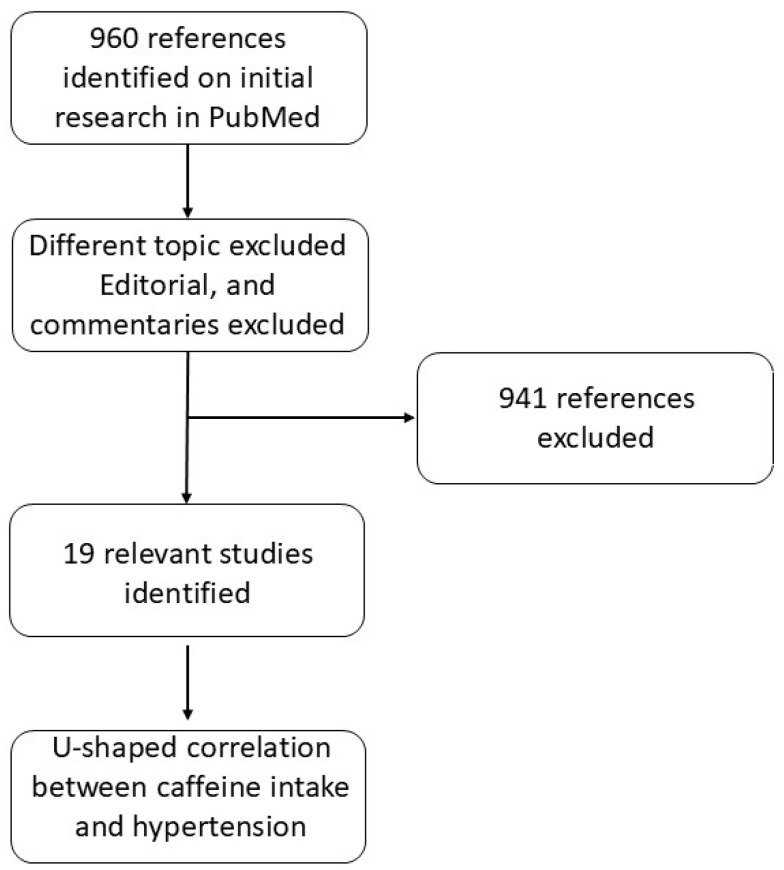
Flow diagram showing the number of records identified, screened, and included in the final analysis to assess the correlation between CAF intake and hypertension.

Coffee intake was associated with a higher risk of CVD mortality in patients with grade 2–3 hypertension with multivariable hazard ratios (95% confidence interval) of CVD mortality that were 0.98 (0.67–1.43) for <1 cup/day, 0.74 (0.37–1.46) for 1 cup/day, and 2.05 (1.17–3.59) for ≥2 cups/day, compared with non-coffee drinkers. Notably, no correlation emerged between coffee consumption and cardiovascular mortality in subjects with normal blood pressure or grade 1 hypertension [81]. Chen et al. evaluated the long-term health effects of CAF in 6076 elderly hypertensive patients from the National Health and Nutrition Examination Survey 2003–2018 with a follow-up of 6.86 ± 0.12 years. Hypertension was defined as being measured with SBP ≥ 140 mmHg or/and DBP ≥ 90 mmHg. Throughout this timeframe, there were 2200 recorded fatalities, with 765 of these attributed to cardiovascular etiologies. Patients with CAF intake < 10 mg/day were utilized as a reference. The results obtained showed that patients with moderate caffeine intake (200 to <300 mg/day) had a lower risk of all-cause (HR 0.70, 95% CI 0.56–0.87) and cardiovascular (HR 0.55, 95% CI 0.39–0.77) mortality. The benefit of reducing all-cause mortality risk was significant in female patients (HR 0.65, 95% CI 0.50–0.85) and well-controlled blood pressure patients (HR 0.63, 95% CI 0.46–0.87). On the contrary, the benefit of reducing all-cause mortality risk was not significant in male patients or patients with poorly self-controlled blood pressure. Moreover, non-linear relationship analysis demonstrated that average caffeine intake displayed the lowest HR of all-cause (non-linear *p* = 0.022) and cardiovascular mortality (non-linear *p* = 0.032) [82]. Rhee et al. analyzed the impact of CAF intake on mean blood pressure and incident hypertension in 29,985 postmenopausal normotensive women. Incident hypertension was defined by using an average SBP ≥ 140 mmHg or an average DBP ≥ 90 mmHg. CAF administration (196 mg/day) was not associated with mean systolic or diastolic blood pressure, as well as coffee consumption up to 2–3 cups/day. Decaffeinated coffee intake was associated with a modest decrease in mean diastolic blood pressure, although clinically irrelevant, while it was not associated with mean systolic blood pressure. Thus, the authors concluded that caffeinated coffee, decaffeinated coffee, and CAF are not associated with the risk of incident hypertension [83]. It has been hypothesized that a relationship exists between psychosocial stress and hypertension [84]. In order to explore this hypothesis, Shepard et al. performed a randomized double-blind crossover trial involving 31 male medical students who were regular consumers of CAF. The cohort included 20 subjects considered at low risk for hypertension due to negative parental history and all screening blood pressure < 125/78 mmHg, and 11 subjects at high risk for hypertension, following epidemiologic criteria such as positive parental history and average screening blood pressure between 125/78 and 139/89 mmHg. Cortisol levels and ambulatory blood pressure were assessed with and without caffeine during conditions of low work stress (lectures) and high work stress (exams). CAF intake and exam stress increased cortisol levels in both groups. Blood pressure increased with CAF or exam stress in both groups. Interestingly, the combination of stress and CAF resulted in additive increases in blood pressure (Low Risk + 9/5 mmHg, High Risk + 10/6 mmHg). Furthermore, 46% of high-risk subjects experienced mean systolic blood pressure ≥ 140 mmHg after CAF intake. Although the study involved a limited number of participants, these findings suggest that individuals at high risk for hypertension should consider refraining from caffeinated beverages and that recent CAF consumption should be assessed in patients undergoing blood pressure evaluation for the diagnosis of hypertension [85]. Another study investigated the effects of CAF and psychological stress on CVD markers in 52 healthy subjects (26 men and 26 women) with a confirmed family history of hypertension. The study examined stress reactivity following CAF intake (3.3 mg/kg). CAF administration resulted in increased systolic blood pressure during stress response and delayed recovery. Interestingly, stress interacted with CAF and sex-altering cortisol, fibrinogen, and systolic blood pressure but not the C-reactive protein level, suggesting sex-specific pathways that associate caffeine with CVD [86]. Analogously, a multicenter prospective study performed by Miranda et al. involved 8780 normotensive subjects who were monitored during a mean follow-up of 3.9 years. Coffee intake was divided into four subgroups based on cups number/day: never/almost never, ≤1, 1–3, and >3.90%; subjects drank coffee, and the median total coffee intake was 150 mL/day. A total of 1285 subjects developed hypertension. Hypertension was defined as a systolic blood pressure of 140 mmHg or a diastolic blood pressure of 90 mmHg, the use of antihypertensive drug treatment, or both. Interestingly, compared to participants, the risk of hypertension was lower for subjects consuming 1–3 cups/day compared to non-coffee drinkers [87]. It has been hypothesized that genotype variations may influence the response to CAF, as it occurs for pharmacological response. A recent study investigated the association of coffee consumption, the rs762551 polymorphism, and hypertension in a cohort of 19,133 subjects. The results indicated that coffee intake for subjects carrying the cytochrome P450 1A2 rs762551 AC + CC genotype was associated with a significantly lower risk of hypertension [88]. A recent study by Liu et al. involved 98,765 subjects and examined the association of CAF with new-onset hypertension, and investigated whether the genetic variation in CAF metabolism may impact this association. Interestingly, the results showed a U-shaped association between unsweetened coffee consumption and new-onset hypertension, with a 14% to 18% reduction in hypertension risk at >1 to ≤4 cups/day, while no association was reported for sweetened coffee intake and the risk of new-onset hypertension. Furthermore, a strong inverse association was found between the moderate consumption of unsweetened coffee and new-onset hypertension in subjects with a higher C-reactive protein level. Finally, individual genetic variations in CAF metabolism did not significantly affect the association [89]. Thus, the authors concluded that the moderate consumption of unsweetened coffee may be associated with a lower risk of hypertension. Wang et al. investigated the association between coffee intake and the risk of dementia in subjects with hypertension utilizing Cox proportional risk modeling with 453,913 participants from a UK biobank. In this study, the baseline hypertensive population was defined by meeting one of the following criteria: self-reported doctor diagnosis of hypertension; self-reported use of antihypertensive medication; measured systolic blood pressure ≥ 140 mmHg or diastolic blood pressure ≥ 90 mmHg. The results showed a U-shaped relationship between caffeine intake and the risk of developing dementia in the hypertensive subjects. Interestingly, hypertensive patients who drank 0.5–1 cup/day displayed the lowest risk of dementia [90]. The U-shaped correlation between CAF and hypertension is highlighted in Figure 3. Sturm et al. investigated the association between CAF consumption and markers of childhood cardiometabolic risk, assuming that high CAF consumption could be associated with elevated blood pressure. Hypertensive blood pressure was defined as SBP ≥ 130 mmHg or DBP ≥ 80 mmHg, and elevated blood pressure was defined as SBP ranging from 120 to 129 mmHg and DBP < 80 mmHg. The study involved a cohort of 3010 patients aged 13–17 with equal distribution between males and females. In fully adjusted regression models, higher CAF consumption was associated with alterations in blood pressure (OR = 0.78, 95%CI [0.52,1.16], *p* = 0.21), dyslipidemia (OR = 0.91, 95%CI [0.65,1.27], *p* = 0.57), glomerular hyperfiltration (OR = 1.01, 95%CI [0.60,1.71], *p* = 0.96), albuminuria (OR = 0.94, 95%CI [0.45,1.98], *p* = 0.87), or insulin resistance (OR = 1.15, 95%CI [0.85,1.56], *p* = 0.36) [91]. Yao et al. explored the association between CAF consumption and all-cause mortality and CVD mortality in 18,914 subjects with pre-diabetes and diabetes from the National Health and Nutrition Examination Survey (NHANES) 2003–2018. Weighted Cox proportional hazard regression models were utilized to assess the HR and 95% CI for all-cause mortality and CVD mortality. The results showed that increased CAF intake was associated with reduced all-cause mortality in pre-diabetic and diabetic subjects. Moreover, individuals with pre-diabetes displayed a significant negative correlation between CAF intake and CVD events [92]. A study performed by Al-Shebel et al. investigated the impact of CAF intake on blood pressure in athletic and non-athletic women involving 30 subjects aged 18–30 years. No significant alterations in diastolic or systolic blood pressure were detected between athletes and non-athletes, thus indicating that CAF consumption does not significantly affect blood pressure in either athletic or non-athletic women [93]. In a recent study, Wang et al. analyzed a cohort of 12,093 US adult subjects to assess a correlation between CAF consumption and mortality in hypertensive individuals. CAF consumption was divided into five groups ranging from no intake up to >400 mg/day. Using multivariable-adjusted Cox proportional hazard models, all CAF consumers had lower all-cause mortality compared with no intake, especially in the group characterized by >300 to ≤400 mg/day intake (HR 0.71, 95% CI 0.60–0.84), while for CVD, mortality decreased only at >400 mg/day (HR 0.63, 95% CI 0.47–0.85). Thus, the data suggest that moderate CAF intake may be beneficial for hypertensive patients [94]. The impact of caffeine on blood pressure is not straightforward and is influenced by several factors, including stress levels, genetic differences, smoking habits, and the presence of hypertension.

**Table 1:** Studies investigating the effects of CAF on hypertension. ↓: downregulation; ↑: upregulation.

Type of CVD	Subjects of the Study	Source of CAF	Main Findings	Limitations	Year	Reference
Hypertension	11 groups of 6 rats	CAF (5–25 mg/kg) and caffeic acid (5–25 mg/kg) administrated orally by gavage	Anti-hypertensive effect of CAF. Systolic blood pressure ↓ ACE activity ↓ Arginase activity ↓ NO levels ↑ MDA levels ↓	Small size group of rats	2021	[75]
Hypertension	1010 human subjects (meta-analysis)	Coffee (725 mL/day) or CAF (410 mg/day)	Systolic pressure ↑	None	2005	[76]
Hypertension	63,257 human subjects (aged 45–74 years)	Daily coffee and caffeinated products consumption (food-frequency questionnaire)	1 cup of coffe/week or more than 2 cups/day reduced risk of hypertension compared to consuming 1 cup of coffee per day	Other ingredients in coffee may offset the effect of CAF	2018	[77]
Hypertension	196,256 human subjects (meta-analysis)	Daily coffee and caffeinated products consumption (food-frequency questionnaire)	Moderate coffee intake is not associated with higher risk of hypertension	A cause-effect relationship between coffee consumption and risk of hypertension cannot be stated based on the evidence available	2019	[78]
Hypertension	48 human subjects (men aged 20–39 years)	CAF (3.3 mg/kg)	Diastolic blood pressure ↑ in patients with borderline hypertension	Small-size cohort groups. Gender biased.	1996	[79]
Hypertension	182 human subjects (men aged 25–40 years)	CAF (3.3 mg/kg, average 260 mg/person or fixed dose of 250 mg)	No impact on blood pressure in non-hypertensive subjects;Blood pressure in patients at stage 1 hypertension ↑	Gender biased	2000	[80]
Hypertension	Human subjects (46,395 men and 64,190 women aged 40 to 79 years)	Daily coffee and caffeinated products consumption (food-frequency questionnaire)	CVD mortality in patients with grade 2–3 hypertension ↑	Other ingredients in coffee/tea may offset the effect of CAF	2023	[81]
Hypertension	6076 humans (elderly hypertensive patients aged aged ≥65 years)	Daily coffee and caffeinated products consumption (food-frequency questionnaire)	Risk of all-cause and cardiovascular mortality in patients with moderate CAF intake ↓	Cohort limited to US country.	2022	[82]
Hypertension	29,985 human subjects (post-menopausal normotensive women aged 50–79 years old)	Daily coffee and caffeinated products consumption (food-frequency questionnaire)	Caffeinated coffee, decaffeinated coffee, and CAF are not associated with risk of incident hypertension	Other ingredients in coffee/tea may offset the effect of CAF	2016	[83]
Hypertension	31 human subjects (men): 20 subjects at low risk and 11 at high risk for hypertension	CAF (3.3-mg/kg)	CAF + stress increased cortisol levels and blood pressure; CAF intake increased mean systolic blood pressure in 46% of high risk subjects	Small-size cohort groups. Gender biased.	2000	[85]
Hypertension	52 human subjects (26 men and 26 women aged 18–29) with family history of hypertension	CAF (3.3-mg/kg)	Systolic blood pressure during stress response ↑; Stress interacted with CAF and sex altering cortisolm fibrinogen, systolic blood pressure	Small-size cohort groups.	2013	[86]
Hypertension	8780 human subjects	Daily coffee and caffeinated products consumption (food-frequency questionnaire)	Risk of hypertention was lower for subjects consuming 1–3 cups/day compared to non-coffee drinkers	Self-reported data on dietary intake and coffee consumption may have resulted in some misclassification and residual confounding	2021	[87]
Hypertension	19,133 human subjects (Taiwanese adults aged 30–70)	Daily coffee and caffeinated products consumption (food-frequency questionnaire)	Subject carrying cytochrome P450 1°2 rs762551 AC + CC genotype was associated with lower risk of hypertension	Cohort limited to Taiwan country	2021	[88]
Hypertension	98,765 human subjects (aged 40–69)	Daily coffee and caffeinated products consumption (food-frequency questionnaire)	Moderate consumption of unsweetened was associated with a lower risk of hypertension	Comorbidities that accompany a new diagnosis of hypertension were not considered	2024	[89]
Hypertension	453,913 human subjects (207,324 men and 246,589 women aged 39–74)	Daily coffee and caffeinated products consumption (food-frequency questionnaire); assumption of 75 mg of CAF/cup of coffee and 40 mg of CAF/cup of tea	Hypertensive patients who drank 0.5–1 cup/day displayed the lower risk of dementia	Self-report of coffee and tea consumption at baseline may be subject to information bias	2024	[90]
Hypertension	3010 human subjects (aged 13–17 with equal distribution between male and female)	Daily coffee and caffeinated products consumption (food-frequency questionnaire)	CAF consumption was not associated with alterations in blood pressure, dyslipidemia, glomerular hyperfiltration, albuminuria, or insulin resistance	The study was based on single-day recall questionnaires. Lack of longitudinal data on the participants in the study. No differentiation between different sources of CAF intake.	2024	[91]
Hypertension	18,914 human subjects (with pre-diabetes and diabetes, aged >20; average age 54.8 years; 9746 men, 9168 women)	24-h dietary recall interviews	Pre-diabetic and diabetic subjects: increased CAF intake → reduced all-cause mortality; Pre-diabetic individuals: significant negative correlation between CAF intake and CVD events.	CAF intake levels were self-reported at baseline, which may result in a different level over long-term follow-up	2024	[92]
Hypertension	30 humans (athletic and non-athletic women, aged 18–30 years)	CAF (80–120 mg)	CAF consumption does not significantly affect blood pressure in either athletic or non-athletic women	Small-size cohort groups. Gender biased.	2024	[93]
Hypertension	12,093 human subjects (5687 men, 6406 women)	24-h dietary recall interviews; CAF (100–400 mg/day)	Moderate CAF intake may be beneficial for hypertensive patients	CAF intake was assessed based on a single day of the interview, which may not accurately represent the long-term intake patterns	2024	[94]

### 3.2. Arrhythmia

Mehta et al. investigated the arrhythmogenic effect of CAF in canine model. The CAF was administered intravenously to perform 51 experiments on 13 anesthetized dogs in 3 different doses (low, medium, high). The results demonstrated a dose-dependent arrhythmogenicity of CAF, although the low dose mainly triggered benign arrhythmias due to vagal stimulation, while higher doses induced more severe arrhythmias, including ventricular tachycardia, atrial fibrillation, and multifocal ventricular premature contractions [95]. Strubelt et al. explored the harmful effects of intravenous infusion of caffeine-sodium salicylate (15 mg/kg/min) in rats. The results of this study demonstrated that CAF triggers sinus tachycardia and ectopic beats in the heart of rats, resulting in fatal ventricular fibrillation in all animals by 66.9 +/− 3.1 min [96]. Ishida et al. further studied the ability of caffeine at a dose ranging from 0.3 to 1.0 mg/kg/min to generate triggered ventricular arrhythmias in rabbits in vivo. The results obtained revealed that CAF administration was correlated with an increased risk of ventricular tachycardia [97]. A large study involving 33,638 women was conducted by Conen et al. in order to clarify the association between CAF intake and arrhythmia. All subjects were free of CVD and atrial fibrillation at baseline and were prospectively followed for incident atrial fibrillation from 1993 to 2009. In this large cohort study, high caffeine consumption was not associated with an increased risk of incident atrial fibrillation [98]. Mostofsky et al. performed a study based on 57,053 Danish subjects (27,178 males and 29,875 females) aged 50–64 years. All individuals provided information on coffee intake via food-frequency questionnaires at baseline, and incident atrial fibrillation was detected using nationwide registries. During a median follow-up of 13.5 years, 3415 events of atrial fibrillation were identified. Compared with the controls (no coffee intake), coffee consumption was inversely correlated with atrial fibrillation occurrence: multivariable-adjusted HRs of 0.93 (95% CI 0.74–1.15) for more than none to <1 cup/day, 0.88 (95% CI 0.71–1.10) for 1 cup/day, 0.86 (95% CI 0.71–1.04) for 2–3 cups/day, 0.84 (95% CI 0.69–1.02) for 4–5 cups/day, 0.79 (95% CI 0.64–0.98) for 6–7 cups/day, and 0.79 (95% CI 0.63–1.00) for >7 cups/day (p-linear trend = 0.02). Thus, the authors concluded that higher levels of coffee intake were associated with a lower rate of episodes of atrial fibrillation [99]. Larsson et al. investigated the association between coffee intake and the incidence of atrial fibrillation in two prospective cohorts and summarized evidence through a meta-analysis. The cohorts included 41,881 men and 34,594 women who provided information on coffee intake in 1997 and were followed up for 12 years. Incident cases of atrial fibrillation were detected using nationwide registries. The results revealed that coffee intake was not associated with atrial fibrillation incidence. Similar findings resulted from the meta-analysis, including six cohort studies with a total of 10,406 patients with atrial fibrillation among 248,910 subjects [100]. Another cohort study involving 130,054 subjects was conducted by Klatsky et al., which evidenced an inverse association between coffee and CAF intake and hospitalization for arrhythmias, suggesting that it is unlikely that moderate CAF intake may increase arrhythmia risk [101]. Dixit et al. performed a cohort study involving 1416 subjects to evaluate dietary patterns and quantify cardiac ectopy using 24-h Holter monitoring; no relationship was found between the chronic consumption of caffeinated products and ectopy [102]. A double-blinded randomized clinical trial with a crossover design was conducted by Zuchinali et al. involving 51 patients with moderate-to-severe left ventricular systolic dysfunction, who received 500 mg of caffeine during a 5-h protocol. No significant association was observed between caffeine consumption and the occurrence of ventricular or supraventricular premature beats. Consequently, the authors concluded that the acute intake of high doses of caffeine did not provoke arrhythmias in patients with systolic heart failure who are at an elevated risk for ventricular arrhythmias [103]. Posch et al. conducted a study in which 101 patients presenting for regadenoson stress myocardial perfusion imaging were asked to provide information about CAF daily intake. No significant association between the use of various quantities of caffeine (ranging from 200 mg/day up to >400 mg/day) and arrhythmia was detected [104]. Frost et al. conducted a study involving 47,949 individuals that prospectively evaluated the association between daily CAF intake and the risk of atrial fibrillation or flutter. After a 5-year follow-up period, no significant association between CAF intake and the incident of atrial fibrillation or atrial flutter was detected [105]. A systematic review and meta-analysis of seven studies involving humans and studies involving animals attempted to clarify the relationship between CAF intake and arrhythmias. In humans, the overall relative risk of ventricular premature beats was 1.00 (95% CI 0.94–1.06). Notably, a remarkable mean change of −2.15 mA in the ventricular fibrillation threshold was detected in studies involving animals (95% CI −3.43 to −0.87), which, however, may be the result of excessive doses of CAF (35 mg/kg) that are not regularly consumed on a daily basis by humans [106]. More recent studies have suggested an inverse correlation between moderate CAF intake and the risk of atrial fibrillation. The meta-analysis conducted by Bazal et al. involved two Spanish cohort studies, including 18,983 and 6479 subjects. Overall, 97 and 250 cases of atrial fibrillation, respectively, were diagnosed during a median follow-up period of 10.3 and 4.4 years. Interestingly, while no association was identified for higher levels of caffeinated coffee intake (>1 cup per day, HR = 0.79 (95% CI 0.49–1.28), average levels (1–7 cups/week) were found to be associated with a reduction in atrial fibrillation risk [107]. Chieng et al. analyzed the long-term effects of decaffeinated, ground, and instant coffee on incident arrhythmia, CVD, and mortality. This study involved a total of 449,563 subjects (median age 58 years) that were followed for 12.5 ± 0.7 years. All coffee types significantly reduced CVD risk, with the lowest risk associated with 2–3 cups/day for decaffeinated coffee (*p* = 0.0093); instant coffee (*p* < 0.000); and ground coffee (*p* < 0.0001). Interestingly, all coffee subtypes triggered a significant reduction of all-cause death, with the maximum risk drop observed for a daily consumption of 2–3 cups of ground coffee (HR 0.73, 95% CI 0.69–0.78, *p* < 0.0001), decaffeinated coffee (HR 0.86, 95% CI 0.81–0.91, *p* < 0.0001), and instant coffee (HR 0.89, 95% CI 0.86–0.93, *p* < 0.0001). Notably, conversely to decaffeinated coffee, 2–3 instant coffee cups/day (HR 0.88, 95% CI 0.85–0.92, *p* < 0.0001) and 4–5 ground coffee cups/day (HR 0.83, 95% CI 0.76–0.91, *p* < 0.0001) induced a significant decrease in arrhythmia incidence [108]. Iten et al. investigated the association between coffee intake and major cardiovascular events in atrial fibrillation patients. Daily coffee intake was associated with a 23% lower hazard for major cardiovascular events compared to irregular consumption (HR 0.77, 95% CI 0.66–0.89). Moderate coffee intake (2–3 cups/day) resulted in the lowest hazard for major cardiovascular events compared to patients with irregular coffee consumption (HR 0.74, 95% CI 0.63–0.87) [109]. Recently, a study including 449,563 UK Biobank participants (median age 58 years) without any cardiovascular problems at enrollment reported that 4–5 cups/day of ground coffee was associated with a significant reduction in incident arrhythmia, including atrial fibrillation (HR 0.83, 95% CI 0.76–0.91, *p* < 0.0001), as well as 2–3 cups/day of instant coffee (HR, 0.88, 95% CI 0.85–0.92, *p* < 0.0001), thus indicating that mild-to-moderate consumption of coffee decreases the risk of CVD and death, and that coffee intake should not be discouraged by physicians [110].

### 3.3. Dyslipidemia

Dyslipidemia is a metabolic disorder characterized by aberrantly high levels of total cholesterol, triglycerides, phospholipids, and low-density lipoproteins (LDLs), and decreased high-density lipoproteins (HDLs). Dyslipidemia plays a crucial role in CVDs, such as hypertension, atherosclerosis, and acute coronary syndrome (ACS), being a significant mortality cause and a heavy burden for healthcare systems [111,112,113,114]. Choi et al. performed a study utilizing 96 rats fed a control diet with coffee (120 mg freeze-dried instant coffee/100 g body weight/day). Coffee administration induced a significant increase in triglyceride level and lowered HDL cholesterol levels compared with the control [115]. Banitalebi et al. conducted a randomized placebo-controlled clinical trial involving 60 obese female subjects, which revealed that green coffee bean extract with elastic resistance band training resulted in significantly decreased levels of total cholesterol [116]. Du et al. performed a systematic review and meta-analysis of 12 randomized controlled trials, revealing that there is a significant association between coffee intake and increased serum levels of triglycerides, total cholesterol, and LDL, while no effect was detected on HDL levels. Thus, the authors concluded that coffee consumption may be associated with an elevated risk of dyslipidemia and CVDs, suggesting that limiting coffee intake could be a strategy for the prevention of dyslipidemia [117]. Similarly, Cai et al. also performed a systematic review and meta-analysis of 12 studies involving a total of 1017 subjects. In line with previous meta-analyses, this study also revealed that coffee intake for 45 days led to an increase of 8.1 mg/dL in total cholesterol, 5.4 mg/dL for LDL, and 12.6 mg/dL for triglycerides. Interestingly, individuals affected by hyperlipidemia were also more inclined to coffee-induced dyslipidemia effects, and a meta-regression analysis showed a positive dose–response association between coffee consumption and total cholesterol, LDL, and triglyceride level [118]. Petrovic et al. investigated the association between methylxanthines as caffeine-deriving metabolites and the plasma lipid level using population-based data collected from Belgium (1987 subjects) and Switzerland (990 subjects). This study revealed that plasma and urinary CAF, paraxanthine, and theophylline were positively associated with plasma lipids. This effect could be the consequence of the sympathomimetic function of methylxanthines, being responsible for the attenuated beneficial effects of CAF intake [119]. Han et al. investigated the associations of coffee consumption with dyslipidemia risk depending on genetic variants in the adenosine receptors (ADORA). The results of this study revealed an inverse association between coffee intake and dyslipidemia incidence in female individuals and that these positive effects are genotype-dependent. Indeed, female subjects carrying the minor alleles of ADORA1 rs10800901, ADORA2B rs2779212, and ADORA3 rs2786967 receive greater protective effects from coffee intake against dyslipidemia, while male subjects carrying the minor allele ADORA3A rs3393 display a lower risk [120]. A recent study performed by Larsson et al. explored the potential causal effects of plasma CAF levels on adiposity, type-2 diabetes, and major CVDs, utilizing a two-sample Mendelian randomization design with summary genetic data. The results of this study revealed that high plasma CAF levels may decrease adiposity and type-2 diabetes risk [121]. All of the abovementioned studies are summarized in Table 2. The evidence collected so far suggests that caffeine intake might be able to increase total cholesterol. However, further studies are required in order to verify this claim.

### 3.4. Acute Coronary Syndrome

Acute coronary syndrome (ACS) occurs due to reduced blood flow in coronary arteries, resulting in the impaired function of part of the heart muscle, and is the most common clinical manifestation of cardiac disease. ACS arises as a consequence of atherosclerotic plaque formations in coronary arteries over time, which can lead to artery occlusion and impaired endothelial function [122,123]. Atherosclerotic plaques progressively widen for many years, and a crack event is responsible for ACS [124]. It has been hypothesized that CAF might contribute to ACS since several cases of ACS following CAF intake have been described (Table 2). Richardson et al. conducted a randomized controlled trial with 103 subjects affected by cardiac autonomic dysfunction post ST-segment elevation myocardial infarction, which revealed that the intake of 352.5 ± 90 mg of CAF can be considered to be safe regarding cardiovascular adverse effects [125]. A prospective study performed by Miranda et al. analyzed the association between coffee intake and all-cause mortality in patients with a prior ACS, utilizing a cohort of 928 individuals. The results obtained demonstrated that a moderate coffee intake was inversely correlated with total mortality; for 1–2 cups/day HR 0.13, 95% CI: 0.06–0.29; for 2–3 cups/day HR 0.22, 95% CI: 0.13–0.39. The subjects with higher coffee consumption (>3 cups/day) displayed a positive association with mortality (HR 2.12, 95% CI: 1.06–4.24) [126]. A systematic review and meta-analysis performed by Brown et al. assessed the association between coffee consumption and mortality in patients with a history of acute myocardial infarction. This study demonstrated that 1–2 cups/day was associated with a risk ratio of 0.79 (95% CI 0.66–0.94, *p* = 0.008), while a higher intake (>2 cups/day) was associated with a risk ratio of 0.54 (95% CI 0.45–0.65, *p* = 0.00001) compared with non-coffee drinkers. Furthermore, heavy coffee drinkers were associated with a risk ratio of 0.69 (95% CI: 0.58–0.83, *p* < 0.0001) compared to light coffee drinkers [127]. The clinical trial TROCADERO by Lindholm et al. hypothesized that CAF could ameliorate ticagrelor-related dyspnea by theophylline, which is an antagonist of adenosine [128]. Furthermore, Surma et al. have recently reported that, despite the controversial results of clinical studies, tea and coffee display the potential to reduce the expression of inflammatory biomarkers and may be protective against CVDs [129]. In line with this assumption, Chieng et al. also recommend that 2–3 cups/day may reduce the risk of coronary heart disease, arrhythmia, heart failure, and cardiovascular death; thus, coffee and tea consumption should be part of a healthy lifestyle [130].

### 3.5. Angina Pectoris

Angina pectoris, also known as stable angina, is a symptom of myocardial ischemia. Stable angina is characterized by chest discomfort and is the first manifestation of underlying coronary disease [131,132]. Several studies have evaluated the impact of CAF intake on chronic stable angina (Table 2). Naderali et al. demonstrated that, in rats, CAF exposure boosts the release of prostacyclin (PGI2), which could be responsible for the beneficial effect of CAF in angina [133]. A clinical trial performed by Piters et al. evaluated the acute effects of coffee on exercise-induced angina in a cohort of 17 male subjects with coronary artery disease. Furthermore, 1 cup/day intake increased exercise duration to 8% until angina onset, while for 2 cups/day, the increase was up to 12%. Moreover, the rate pressure product and the extent of ST-segment depression at angina onset were found to be analogous both after normal coffee intake and decaffeinated coffee [134]. Thus, taken together, there is some evidence that a low daily amount of coffee intake may participate in the time to angina onset in individuals with chronic stable angina.

### 3.6. Heart Failure

Heart failure (HF) is a complex syndrome caused by an impaired ability of the heart to pump blood and oxygen to support the body’s needs and is the consequence of structural or functional heart abnormalities [135,136,137]. Several animal and human studies have evaluated the impact of CAF intake on HF. Tofovic et al. reported that caffeine enhances basal renin secretion by blocking intrarenal adenosine receptors. Moreover, in the case of increased sympathetic activity, CAF boosts renin release in part through a blockade of brain adenosine receptors, which results in enhanced central sympathetic tone [138,139]. The same research group performed a subsequent study utilizing rats to assess the consequences of acute and short-term caffeine intake on heart performance. While 10 mg/kg caffeine followed by 150 g/min over 40 min did not affect cardiac contractility, it enhanced both the heart rate and left-ventricular peak systolic pressure and enhanced plasmatic norepinephrine, epinephrine, and renin activity levels. Short-term CAF intake for 10 days did not result in significant alterations in cardiac time-pressure variables or hemodynamic/renal excretory function parameters, although it was able to enhance renal renin secretion [140]. Notarius et al. investigated the impact of CAF on exercise duration in HF in a double-blind clinical trial where 10 subjects affected by HF were administered 4 mg/kg of CAF on 2 different days. Data obtained revealed that caffeine intake can enhance both exercise duration and performance, thus suggesting that a moderate CAF intake could be beneficial for HF patients [141]. Bodar et al. performed a large-scale, long-term prospective study to evaluate the relation between coffee consumption and HF, utilizing a cohort of 20,433 male subjects (mean age 66.4 ± 9.2 years). Coffee intake was monitored utilizing a semi-quantitative food frequency questionnaire, and the incidence of HF was collected based on annual self-reports and through a review of medical records. During a mean follow-up of 9.3 years, a total of 901 subjects were diagnosed with HF, but data analysis demonstrated no significant correlation between coffee (*p* = 0.47) and CAF (*p* = 0.34) intake and the risk of HF [142]. In the same vein as these results, a Mendelian randomization study performed by Oort et al. reported no associations of coffee intake and HF [143]. Mostofsky et al. performed a meta-analysis including 140,220 subjects, which revealed that 4 cups/day intake of coffee seems to be protective against HF [144]. Tikhonoff et al. investigated the prognostic effects of being over the cut-off of all-source dietary CAF in a cohort of 1668 subjects. Prognostic cut-off values utilized in the study were >230 mg/day for heart failure and 280 mg/day for arrhythmic events. The results of this study demonstrated that men introducing > 230 mg/day caffeine present a reduced risk of heart failure, while CAF intake > 280 mg/day can reduce the risk of cerebrovascular events and arrhythmic events [145].

### 3.7. Other CVDs

Yüksel et al. evaluated the impact of CAF administration on cardiac health in 60 rats with propylthiouracil-induced hypothyroidism. The obtained results showed that administering CAF alongside or after propylthiouracil treatment could improve thyroid and cardiac irregularities, suggesting that CAF displays the potential to mitigate the harmful effects of hypothyroidism on thyroid and heart health [146]. A recent study by Parladori et al. investigated the influence of CAF maintenance on cardiovascular and cerebrovascular hemodynamics, utilizing a cohort of 77 infants (mean age 29.3 ± 2.5 weeks). CAF administration was associated with increased systemic vascular resistance (*p* = 0.004) and more negative tissue oxygenation-heart rate reactivity index values (*p* = 0.022), suggesting an ameliorated cerebrovascular reactivity. Thus, the authors concluded that CAF administration at the maintenance dosage during postnatal transition may be beneficial, likely due to the CAF-mediated inhibition of adenosine receptors [147]. Parks et al. reported that CAF intake (4 mg/kg) does not induce any alterations in resistance exercise performance or have negative effects on the cardiovascular system [148]. An interesting study performed by Fan et al. explored the effect of coffee intake on abdominal aortic calcification among 2548 adult subjects (aged 40–79) with and without hypertension, diabetes, and CVDs. Heavy coffee consumption (>390 g/day) was associated with higher abdominal aortic calcification scores among individuals with hypertension, diabetes, and CVDs, whereas no significant association was detected among healthy participants; thus, the authors concluded that coffee consumption should be limited in patients with hypertension, diabetes, and CVDs in order to reduce the rate of abdominal aortic calcification [149].

Liver sinusoidal endothelial cells (LSECs) line the fenestrated wall of hepatic sinusoids and exert a key role in regulating and surveying the trafficking of molecules between liver parenchyma and blood [150]. Mao et al. evaluated the effect of CAF on cultured primary rat LSEC fenestrated morphology. LSEC porosity was affected only by high CAF amounts. which also changed the fenestration distribution towards smaller pores, and a dose-dependent rise in fenestration number was detected upon CAF treatment, suggesting a potential CAF-treatment strategy to counteract an age-related reduction of LSECs porosity [151].

**Table 2:** Studies investigating CAF effects on other CVDs. ↓: downregulation; ↑: upregulation.

Type of CVD	Subjects of the Study	Source of CAF	Main Findings	Limitations	Year	Reference
Arrhythmia	13 dogs	CAF	Dose-dependent arrhythmogenecity of CAF	Small-size group To be confirmed in human clinical trial.	1997	[95]
Arrhythmia	14 male wistar rats	CAF-sodium salicylate (15 mg/kg/min)	CAF triggered sinus tachycardia and ectopic beats of heart resulting in fatal ventricular fibrillation	Small-size group To be confirmed in human clinical trial	1999	[96]
Arrhythmia	34 adult male Japanese white rabbits	CAF (0.3 or 1.0 mg/kg per min)	CAF administration was correlated with an increased risk of ventricular tachycardia	To be confirmed in human clinical trial	1996	[97]
Arrhythmia	33,638 women	Daily coffee and caffeinated products consumption (median caffeine intakes across increasing quintiles of caffeine intake were 22, 135, 285, 402, and 656 mg/d, respectively)	High caffeine consumption was not associated with an increased risk of incident atrial fibrillation	Only two measures over years may miss short-term effects; limited to middle-aged, white, female health professionals, affecting generalizability to men or other female populations; limited statistical power to find associations in small beverage subgroups; no ECG screening: possibly missing undetected AF cases; difficult to accurately defining AF onset, potentially introducing small bias if the time of incidence is incorrectly specified	2010	[98]
Arrhythmia	57,053 Danish subjects (27,178 males and 29,875 females) aged 50–64 years	Daily coffee and caffeinated products consumption (food-frequency questionnaire)	Coffee consumption was inversely correlated with atrial fibrillation occurrence	No distinction between impacts of caffeinated or decaffeinated coffee; No information on brewing method or genetic polymorphism; Limited to cases with recorded hospitalizations or deaths for AF	2016	[99]
Arrhythmia	41,881 men and 34,594 women	Daily coffee and caffeinated products consumption (food-frequency questionnaire)	Coffee intake was not associated with atrial fibrillation incidence	AF cases in the cohort are symptomatic, possible bias introduced if patients with with first episode of less seious AF reduced coffe consumption; Assessment of coffee consumption can show some measurement error because it was assessed with self-administered questionnaire and only at baseline: no information on the type of coffee and preparation method	2015	[100]
Arrhythmia	130,054 human subjects	Daily coffee and caffeinated products consumption (food-frequency questionnaire)	Inverse association between coffee and CAF intake and hospitalization for arrhythmias	No data about follow-up coffee use; Incomplete caffeine data: no data about circumstances leading to hospitalization, coffee preparation method, cup size, and time of day for the coffee intake	2011	[101]
Arrhythmia	1416 human subjects (44.1% men, 55.9% women)	Daily coffee and caffeinated products consumption (food-frequency questionnaire)	No relationship between chronic consumption of caffeinated products and ectopy	No evidence of a clinically large effect; Patients self-reporting; absence of total caffeine quantification; no discrimination among amounts of daily consumption	2016	[102]
Arrhythmia	51 patients with moderate-to-severe left ventricular systolic dysfunction (37 men; 14 women, mean age 60.6 years)	CAF (total of 500 mg during a 5-h protocol)	No significant association between CAF intake and the incidence of ventricular and supraventricular premature beats	Small-size cohort groups.	2016	[103]
Arrhythmia	101 patients presenting for regadenoson stress myocardial perfusion imaging	Daily coffee and caffeinated products consumption (food-frequency questionnaire)	No significant association between use of CAF and arrhythmia was detected	Patient self-reporting	2020	[104]
Arrhythmia	47,949 human subjects aged 50–64 years (22,533 men and 25,416 women)	Daily coffee and caffeinated products consumption (food-frequency questionnaire)	No significant association between CAF intake with the incident of atrial fibrillation or atrial flutter was detected	Self-reported data on the consumption of caffeine; caffeine content change across brands	2005	[105]
Arrhythmia	Animals and humans (meta-analysis)	CAF	Increased risk of ventricular premature beats in humans. Mean change of −2.15 mA in ventricular fibrillation threshold was detected in studies involving animals	Effects found in animal studies areprobably the result of excessive caffeine doses that are not consumed on regular daily basis in humans (35 mg/kg)	2016	[106]
Arrhythmia	18,983 and 6479 human subjects (meta-analysis)	Daily coffee and caffeinated products consumption (food-frequency questionnaire)	No association was identified for higher levels of caffeinated coffee intake (>1 cup per day). Average levels (1–7 cups/week) were found to be associated with a reduction in atrial fibrillation risk	Possibility of reverse causality in the association between coffee consumption and AF cannot be excluded	2021	[107]
Arrhythmia	449,563 human subjects(median 58 years, 55.3% females)	Daily coffee and caffeinated products consumption(0, <1, 1, 2–3, 4–5, and >5 cups/day)	Coffee consumption reduced CVD risk and reduction of all-cause death. Decaffeinated coffee decreased arrhythmia incidence.	Participant self-reporting; outcome assessment relied on ICD-10 codes; detection of certain arrhythmias may be missed; predominantly Caucasian population	2022	[108]
Arrhythmia	3835 human patients (Swiss-AF, *n* = 2387; Beat-AF, *n* = 1507)	“daily” and “not-daily” coffee consumers	Daily coffee intake was associated with a 23% lower hazard for major cardiovascular events	The main population of study was of European origin; coffee consumption was self-assessed by the patients; male patients are overrepresented	2024	[109]
Arrhythmia	449,563 human subjects; median age 58 years; 55.3% females, of which 100,510 (22.35%) were controls (non-coffee drinkers)	Daily coffee and caffeinated products consumption (food-frequency questionnaire)	4–5 cups/day of ground coffee was associated with a significant reduction in incident arrhythmia including atrial fibrillation	Coffee consumption was self-reported; ICD-10 codes is susceptible to measurement and reporting errors; population of the study is predominantly Caucasian therefore study conclusions may not be entirely applicable to populations of other ethnicities.	2023	[110]
Dyslipidemia	96 4-week old male, Sprague-Dawley rats	Coffee (average amount of coffee intakes per rat: 0.12 g freeze-dried instant coffee/100 g body weight/d).	Triglycerides ↑ HDL cholesterol ↓	Applying study results to athletes performance is difficult	2013	[115]
Dyslipidemia	60 humans (obese women aged 30–50 years)	Green coffee bean extract (500 mg)	Levels of total cholesterol ↓	Small-size cohort groups. Short duration of the study. Gender biased.	2019	[116]
Dyslipidemia	1182 human subjects (meta-analysis, aged 18–70 years)	Daily coffee consumption	Serum levels of triglycerides ↑ Total cholesterol ↑ LDL ↑	Small sample size; Different type and concentration of coffee; no enough evidence to assess effect of coffee consumption on HDL-C	2020	[117]
Dyslipidemia	1017 human subjects (meta-analysis)	Daily coffee consumption	Total cholesterol ↑ LDL ↑ Triglycerides ↑ Individuals affected by hyperlipidemia were more inclined to coffee-induced dyslipidemia effects	Bias in the meta-analysis of total cholesterol	2012	[118]
Dyslipidemia	Humans (1987 subjects from Belgium and 900 subjects from Swiss; in both studies 53% of subjects were females)	CAF-derived metabolites plasma levels (methylxanthines)	Total cholesterol ↑ LDL ↑ Triglyceride levels ↑	Population biased. No information about source of plasma methylxanthines source	2021	[119]
Dyslipidemia	Human subjects (2527 men and 2371 women)	Daily coffee consumption	Female subjects: minor alleles of ADORA1 rs10800901, ADORA2B rs2779212, and ADORA3 rs2786967 → higher protective effects from coffee intake against dyslipidemia Male subjects: minor allele ADORA3A rs3393 → lower risk	Small-size cohort group; No information regarding amount of CAF consumed; Physical activity could influence the blood lipid profile	2020	[120]
Dyslipidemia	9876 human subjects	Daily coffee consumption	High plasma CAF levels may decrease adiposity and type-2 diabetes risk	Two-sample Mendelian randomization design; causal findings may not be applied to clinical or public health interventions; use of only two SNPs reduced analysis power; results may not be generalisable to non-European populations	2023	[121]
Acute Coronary Syndrome	103 human subjects with acute STEMI (males)	Coffee or decaffeinated coffee (352.5 ± 90 mg (4.7 ± 1.1 cups) of CAF per day and 4.5 ± 1.3 cups per day of decaffeinated beverage)	CAF can be considered to be safe regarding cardiovascular adverse effects	Small-size cohort group; post-STEMI dysrhythmias did not allow for the interpretation of 24-h HRV analyses	2009	[125]
Acute Coronary Syndrome	928 human subjects with acute coronary syndrome	Daily coffee and caffeinated products consumption	Moderate coffee intake was inversely correlated with total mortality	No information about brewing process or coffee type	2021	[126]
Acute Coronary Syndrome	1369 and 1902 human subjects (meta-analysis)	Daily coffee consumption	Intake > 2 cups/day was associated with a risk ratio of 0.54	No information about brewing process or coffee type Publication bias	2016	[127]
Angina Pectoris	17 male rats	CAF (ranging 1 µg–1 mg)	CAF exposure boosts the release of prostacyclin (PGI2) which could be responsible for the beneficial effect of CAF in angina	Small-size group. To be confirmed in human clinical trial	1994	[133]
Angina Pectoris	17 male subjects with coronary artery disease	Daily coffee and caffeinated products consumption	2 cups/day intake increased the exercise duration of 12% until angina onset	Small-size cohort groups. Gender biased.	1985	[134]
Heart Failure	14 male Sprague-Dawley rats	CAF 10 µg/kg/min	CAFenhances basal renin secretion by blocking intrarenal adenosine receptors and, in case of increased sympathetic activity, CAF boosts renin release in part by blockade of brain adenosine receptors, which results in enhanced central sympathetic tone	Small-size cohort groups.	1996	[138,139]
Heart Failure	Seven male, 9-month-old SHHF/Mcc-fa^cp^ rats; Seven 9-month-old SHRs eight 9-month-old normotensive WKY rats; Fifteen aged (14-month-old) male lean SHHF/Mcc-fa^cp^ rats	CAF (10 mg/kg + 150 μg/min over 40 min)	10 mg/kg CAF followed by 150 g/min over 40 min enhanced both heart rate and left-ventricular peak systolic pressure and enhanced plasmatic norepinephrine, epinephrine, and renin activity levels	Small-size cohort group Long-term studies are needed	1999	[140]
Heart Failure	10 human subjects (7 men, 3 women)	CAF (4 mg/kg) intravenously	CAF intake enhanced both exercise duration and performance	Small-size cohort group	2006	[141]
Heart Failure	20,433 men (mean age 66.4)	Daily coffee and caffeinated products consumption (food-frequency questionnaire)	No significant correlation between coffee and CAF intake and the risk of HF	CAF intake was based on participant self-reporting, with the attendant risk of reporting bias	2020	[142]
Heart Failure	140,220 human subjects (meta-analysis)	Daily coffee and caffeinated products consumption	4 cups/day intake of coffee seems to be protective against HF	Other ingredients in coffee may offset the effect of caffeine	2012	[144]
Heart Failure	1668 human subjects (751 men, 917 women, aged 58.8–80.5)	Daily coffee and caffeinated products consumption (food-frequency questionnaire)	Introducing > 230 mg/day CAF present a reduced risk of heart failure, while CAF intake > 280 mg/day can reduce risk of cerebrovascular events and arrhythmic events	CAF intake was based on participant self-reporting, with the attendant risk of reporting bias	2023	[145]
Hypothyroidism	60 female Wistar albino rats	CAF 10 mg/kg/day in water via gavage for 2 months	CAF administration could improve thyroid and cardiac irregularities	Lack of dose dependent observations due to the use of a single concentration	2025	[146]
Systemic vascular resistance	77 infants (<32 weeks gestational age; 39 male and 38 female)	CAF citrate 5 mg/kg	CAF administration was associated with increased systemic vascular resistance and more negative tissue oxygenation-heart rate reactivity index values	Lack of continuous capnography monitoring	2024	[147]
Cardiovascular Health	11 women (mean age 24)	CAF 4 mg/kg	CAF intake did not induce any negative effects on the cardiovascular system	Small-size cohort group	2023	[148]
Abdominal aortic calcification	2548 adult subjects (age mean 58.71)	24-h dietary recall interviews	Heavy coffee consumption (>390 g/day) was associated with higher abdominal aortic calcification scores among individuals with hypertension, diabetes, and CVDs	A causal relationship between coffee consumption and abdominal aortic calcification could not be considered due to the crosssectional study design	2023	[149]
Liver sinusoidal endothelial cells defenestration	Primary rat LSECs	CAF (8 and 150 μg/mL)	LSECs porosity and fenestration distribution was affected by high CAF amounts. A dose-dependent rise in fenestration number was detected upon CAF treatment.	Very high doses of CAF	2023	[151]

## 4. Caffeine Intoxication

Although severe CAF intoxication is rare, it may accidentally occur. Severe intoxication may be fatal, usually due to malignant cardiac arrhythmia. The median lethal dose (LD50) of CAF is estimated to range between 150 and 200 mg/kg, but there are cases of fatal intoxications that occur with doses as low as 57 mg/kg [152]. A systematic review reported that the evidence generally supports that <400 mg caffeine/day intake in healthy adults is not associated with adverse cardiovascular effects. Moreover, this level of intake is also not associated with behavioral, reproductive, developmental, or acute effects, as well as bone status. Moreover, evidence also supports that <300 mg caffeine/day in healthy pregnant women is generally not associated with adverse reproductive and developmental impacts. For children and adolescent populations, the available evidence suggests that 2.5 mg caffeine/kg body weight/day remains an appropriate recommendation [153]. Cabral et al. reported the case of a male student that accidentally consumed 6000 mg of CAF in the form of an energy supplement of CAF. The patient presented with nausea, vomiting, palpitations, dizziness, sinus tachycardia, abdominal tenderness, and clammy skin. Moreover, the arterial blood gases test showed progressively worsening metabolic acidosis accompanied by severe hypokalemia and type B hyperlactacidemia [154]. It has been reported that in patients with migraines, caffeine intake is associated with reduced cerebrovascular reactivity in the posterior circulation, and thus CAF cessation may have positive effects on improving cerebrovascular reactivity [155]. De Souza et al. recently reported that CAF supplementation in athletes is often accompanied by tachycardia/heart palpitations and negative effects on sleep onset [156]. It is noteworthy to underline that excessive CAF intake may be hidden. For instance, herbal medicines or supplements for weight loss may include high CAF content, leading to an underestimated total CAF intake. Moreover, unknown or accidental interactions between CAF and other active molecules in CAF-containing products may contribute to enhancing the risk of side effects [157].

## 5. Conclusions

The present review summarized the available literature regarding preclinical and clinical studies on the effect of CAF in the context of CVDs. Although general evidence supports an overall beneficial effect of CAF in the context of CVDs, there are several limitations that may influence the reproducibility and reliability of the available data regarding the efficacy and safety of caffeine in CVDs. Inconsistencies in dose standardization, mainly due to the use of a precise dose of CAF or generic quantization in coffee cups, may influence the results. We strongly believe that further studies should be performed utilizing caffeine capsules instead of measuring daily cup intake, since the results of the studies could be largely influenced depending on coffee type/breed, the procedure utilized for preparing coffee beverages, and the cup size. For instance, stronger brews are characterized by higher levels of caffeine, antioxidants, and other bioactive molecules that in turn impact cardiovascular health. Furthermore, the duration and regularity of the intake, an unaware CAF intake diet, and the genetic, demographic, and clinical data of patients may also affect the results. However, there is solid evidence that caffeine can be considered safe in individuals without significant CVDs. Further studies should be properly designed, identifying an appropriate cohort size with a precise characterization of other possible factors that could influence the outcome of the study on CVDs.

## Data Availability

No new data were created or analyzed in this study.
